# Stability of gene expression by primary bronchial epithelial cells over increasing passage number

**DOI:** 10.1186/s12890-018-0652-2

**Published:** 2018-05-29

**Authors:** Stephen R. Reeves, Kaitlyn A. Barrow, Maria P. White, Lucille M. Rich, Maryam Naushab, Jason S. Debley

**Affiliations:** 1 Center for Immunity and Immunotherapies, Seattle Children’s Research Institute, Seattle, WA USA; 2 Pulmonary and Sleep Medicine Division, Department of Pediatrics, University of Washington, Seattle, WA USA

**Keywords:** Asthma, Children, Airway remodeling, Epithelial cells

## Abstract

**Background:**

An increasing number of studies using primary human bronchial epithelial cells (BECs) have reported intrinsic differences in the expression of several genes between cells from asthmatic and non-asthmatic donors. The stability of gene expression by primary BECs with increasing cell passage number has not been well characterized.

**Methods:**

To determine if expression by primary BECs from asthmatic and non-asthmatic children of selected genes associated with airway remodeling, innate immune response, immunomodulatory factors, and markers of differentiated airway epithelium, are stable over increasing cell passage number, we studied gene expression patterns in passages 1, 2, 3, 4, and 5 BECs from asthmatic (*n* = 6) and healthy (n = 6) subjects that were differentiated at an air-liquid interface. RNA was harvested from BECs and RT-PCR was performed for TGFβ1, TGFβ2, activin A, FSTL3, MUC5AC, TSLP, IL-33, CXCL10, IFIH1, p63, KT5, TUBB4A, TJP1, OCLN, and FOXJ1.

**Results:**

Expression of TGFβ1, TGFβ2, activin A, FSTL3, MUC5AC, CXCL10, IFIH1, p63, KT5, TUBB4A, TJP1, OCLN, and FOXJ1 by primary BECs from asthmatic and healthy children was stable with no significant differences between passages 1, 2 and 3; however, gene expression at cell passages 4 and 5 was significantly greater and more variable compared to passage 1 BECs for many of these genes. IL-33 and FOXJ1 expression was also stable between passages 1 through 3, however, expression at passages 4 and 5 was significantly lower than by passage 1 BECs. TSLP, p63, and KRT5 expression was stable across BEC passages 1 through 5 for both asthmatic and healthy BECs.

**Conclusions:**

These observations illustrate the importance of using BECs from passage ≤3 when studying gene expression by asthmatic and non-asthmatic primary BECs and characterizing the expression pattern across increasing cell passage number for each new gene studied, as beyond passage 3 genes expressed by primary BECs appear to less accurately model in vivo airway epithelial gene expression.

**Electronic supplementary material:**

The online version of this article (10.1186/s12890-018-0652-2) contains supplementary material, which is available to authorized users.

## Background

Asthma continues to be one of the most prevalent and costly diseases of childhood throughout the world [1]. In recent years, our understanding of asthma pathogenesis has grown to include a central role for bronchial epithelial cells (BECs) in the establishment and maintenance of asthmatic airway disease [2]. Additionally, understanding of the importance of BECs in immune surveillance and coordination of the immune response to infections and environmental antigens has grown to include an important role for BEC-derived cytokines and direct cell-to-cell communication beyond their role in barrier function and innate immunity [3]. As a result, BECs have become the focus of many recent studies aimed to elucidate mechanisms underlying asthma pathogenesis in children [4].

Studying BECs in children with and without asthma presents a unique set of challenges. Unlike studies in adults, obtaining cells via bronchoscopic airway biopsy from children is problematic as most institutional review boards cannot approve such studies in children as the risk of general anesthesia and the lack of direct benefit to the subject precludes such investigations. While transformed and immortalized cell lines exist and are commercially available for in vitro studies, none of the currently available cell lines are derived from pediatric donors, and very little clinical data is provided about the subjects from whom such lines were obtained. Furthermore, immortalized BEC cell lines are variable in their ability to recapitulate the anatomical and physiologic features observed in the in vivo condition [5]. Direct comparisons of airway epithelial behavior in the asthmatic vs. non-asthmatic conditions is critical to furthering our understanding of the pathophysiology of asthma, which therefore requires use of primary airway epithelial cells. In addition, given the recognition that asthma is a complex and heterogeneous disease with multiple endotypes with likely unique aspects driving pathophysiology [6], it is increasingly important that donors of primary epithelial cells used in basic and translational studies be carefully clinically characterized so as to allow investigations of the role of the airway epithelium within unique asthma endotypes.

Given the limitations of in vitro models utilizing cell lines and the difficulty obtaining biopsy specimens in the pediatric population, ex vivo cultures of primary BECs obtained via bronchial brushings have become a useful model in which to perform studies related to the airway epithelium in children [7]. Collection of bronchial epithelial cells using brushings performed through an endotracheal tube while a patient is under general anesthesia (taking advantage of a planned anesthesia for a separate, clinically indicated procedure) have been described and importantly pose minimal additional risk to the subject [7, 8]. While this methodology has been utilized successfully in children and adults for more than a decade and provides a reliable, safe, and relatively non-invasive way to obtain primary BECs [7–10] that can be utilized to support ex vivo studies, the major trade-off is that the number of cells obtained using this methodology is relatively low. In most cases expansion of the cell number in culture is an essential step required to produce sufficient material to conduct experiments.

While the passage of cells in culture poses a theoretical loss of cellular phenotype the further cells are propagated beyond the donor, several studies including work from our lab have demonstrated phenotypic differences in primary cells obtained from children with and without asthma (reviewed by McLellan et al.) suggesting that cell phenotype is preserved at least in the initial 2-3 passages [4]. While there have been anecdotal reports of cells performing poorly in cell cultures at later passages, we are not aware of studies that have rigorously examined gene expression differences that occur in BECs over subsequent passages. In order to better characterize the stability of gene expression by primary BECs over multiple passages, we tested the hypothesis that the expression of a panel of genes involved in airway remodeling, immune regulation, innate immune response, airway epithelial basal cells, ciliogenesis, and epithelial tight junctions would be stable through increasing cell passages in an ex vivo organotypic culture model system using primary BECs obtained from well-phenotyped children with or without asthma that were differentiated at an air-liquid interface (ALI).

## Methods

### Subjects

For this study, we recruited asthmatic and healthy children ages 6-18 years undergoing an elective surgical procedure that required endotracheal intubation and general anesthesia for a clinically indicated procedure. Children with asthma had at least a 1-year history of physician-diagnosed asthma, used a short-acting beta-agonist ≥ twice a month or were taking a daily maintenance medication (inhaled corticosteroid or montelukast), and were born ≥36 weeks gestation. Additionally, children with asthma had one or more of the following atopic features: history of a positive skin prick test or positive radioallergosorbent testing (RAST) for a common aeroallergen, elevated serum IgE (> 100 IU/mL), history of physician-treated allergic rhinitis, history of physician-treated atopic dermatitis. Healthy subjects were born ≥36 weeks gestation, and lacked a history of asthma, reactive airway disease, chronic cough, chronic lung disease, or past treatment with bronchodilators, systemic or inhaled steroids, or oxygen. A detailed medical history was obtained to ensure the subjects met these inclusion criteria.

At the time of anesthesia, a blood sample was drawn from each subject and used to measure total serum IgE and RAST allergen-specific IgE to dust mites (D. farina and *D. pteronyssinus*), cat epithelium, dog epithelium, *Alternaria tenuis*, Aspergillus fumigatus, and timothy grass. At a subsequent follow-up visit the fraction of exhaled nitric oxide (FE_NO_) was measured according to American Thoracic Society (ATS) guidelines using a NIOX MINO nitric oxide analyzer (Aerocrine®, Sweden) [11]. Spirometry was performed using a VMAX® series 2130 spirometer (VIASYS Healthcare, Hong Kong) to quantify forced vital capacity (FVC), forced expiratory volume in 1 s (FEV_1_), and forced expiratory flow between 25 and 75% of FVC (FEF_25-75_) according to ATS guidelines.

### Ethics, consent and permissions

Written consent was obtained from parents of subjects and assent was obtained for children ≥ age 10 years. The work presented in this study was approved by the Seattle Children’s Hospital Institutional Review Board.

### Establishment of bronchial epithelial cell cultures

Following the induction of anesthesia and securing of the endotracheal tube, three samples of BECs were obtained from each subject using 4 mm Harrell® unsheathed bronchoscope cytology brushes (CONMED® Corporation). The brush was inserted through an endotracheal tube, advanced until resistance was felt, and rubbed against the airway surface for 2-3 s as described previously [7, 8]. Cells were seeded onto type I collagen coated T-25 cell culture flasks and proliferated under submerged culture conditions. Cultures were proliferated in a humidified incubator at 37 °C in an atmosphere of 5% CO_2_ in PneumaCult™-Ex bronchial epithelial growth medium (BEGM) (StemCell™ Technologies) containing gentamicin and amphotericin B, and supplemented with penicillin-streptomycin (100 μg/ml; Invitrogen®). Fluconazole (25 μg/mL) was added to P0 medium for the first 96 h, after which medium was aspirated and replaced with medium without fluconazole. Medium was thereafter changed every 48 h until the culture reached ~ 70-90% confluence. All primary BEC lines were screened for mycoplasma using MycoAlert™ PLUS Mycoplasma Detection Kit (Lonza, Inc) and found to be negative.

### Air-liquid Interface (ALI) epithelial cell cultures

BECs were used for these studies at each passage corresponding number. Once cells were ~ 70-90% confluence in flasks, they were trypsinized with 1 mL of 0.025% Trypsin-EDTA and then seeded onto collagen I pre-coated (Collagen Solution, STEMCELL™ Technologies) Corning Costar 12 mm 0.4 μm Transwells® (Corning® Life Sciences) at a concentration of 100,000 cells per transwell. Cells were then kept in submerged culture using BEGM in both the apical and basolateral well chambers for 7 days or until confluent. Once confluent, cells were then changed to Pneumacult™ ALI Medium (StemCell™ Technologies) in the lower basolateral chamber only and the remaining apical media was aspirated. ALI media in the basolateral compartment was changed every other day and cells were differentiated at an ALI for 21 days.

### Study design

**Fig. 1:**
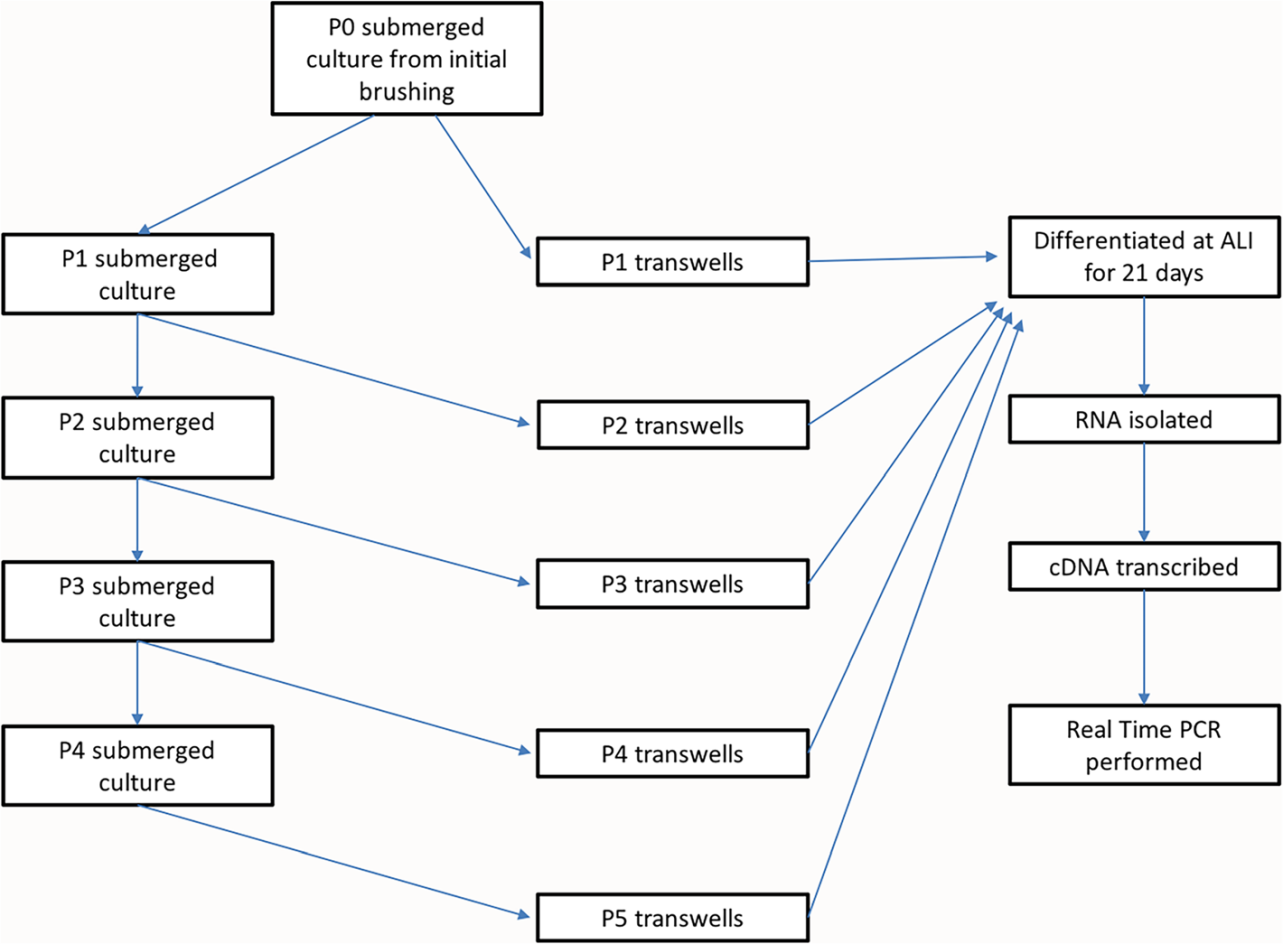
Schematic depicting experimental design for passaging primary bronchial epithelial cells over 5 passages in ex vivo cell culture for gene expression studies

BECs seeded into the initial T25 flasks were designated passage 0 (P0) and allowed to proliferate until cells were ~ 70-90% confluent at which point cells were passaged. One of the three flasks was then passaged into a transwell plate in order to establish P1 ALI cultures for experiments. Another P0 T25 flask was split into 3 additional T25 flasks (also P1) to carry forward into subsequent experiments. The remaining P0 T25 was either preserved in liquid nitrogen or also carried forward for separate studies. This paradigm was carried forward for each passage (P + 1) until reaching ALI cultures up through passage 5 (P5) to conduct experiments (Fig. 1).

### RNA extraction and real-time PCR

Total RNA was isolated from BECs differentiated at an ALI. Three wells from each experimental condition were harvested and pooled to isolate RNA using the RNAqueous kit for total RNA purification from Ambion®-Applied Biosystems (Austin, TX). RNA concentration and quality was determined using a NanoDrop ND-1000 spectrophotometer Thermo Fisher Scientific). RNA samples (1 μg) were reverse-transcribed using the SuperScript® VILO cDNA Synthesis Kit (Life Technologies, Grand Island, NY). Samples were diluted up to a final volume of 100 μl (10 ng/μl). Quantitative real-time PCR was performed using validated TaqMan® probes (Life Technologies) for transforming growth factor beta (TGFβ)1 (Hs00998133_m1), TGFβ2 (Hs00234244_m1), activin A (Hs01081598_m1), follistatin-like-3 (FSTL3, Hs00610505_m1), mucin 5 AC (MUC5AC, Hs01365616_m1), thymic stromal lymphopoietin (TSLP, Hs00263639_m1), interleukin-33 (IL-33, Hs01125942_m1), C-X-C motif chemokine 10 (CXCL10, Hs00171042_m1), interferon induced with helicase C domain (1IFIH1, Hs00223420_m1), p63 (TP63, Hs00978340_m1), cytokeratin 5 (KRT5, Hs00361185_m1), beta-tubulin (TUBB4A, Hs00760066_s1), forkhead box J1 (FOXJ1, Hs00230964_m1), zona occluden-1 (TJP1, Hs01551861_m1), occludin (OCLN, Hs05465837_g1), and glyceraldehyde 3-phosphate dehydrogenase (GAPDH, Hs02758991_g1). Assays were performed using the TaqMan® Fast Advanced Master Mix reagents and accompanying protocol and the Applied Biosystems StepOnePlus™ Real-Time PCR System with StepOne Software v2.2.2 (Life Technologies). The primary quantitative PCR datasets used and/or analyzed for this study are provided as a (Additional file 1: Appendix I).

### Statistical analysis

For clinical parameters, the paired t-test was used for comparisons that were normally distributed within each subject group. For non-normally distributed data, the Wilcoxon signed-rank test was used. For RT-PCR studies, the relative expression of genes were normalized using glyceraldehyde 3-phosphate dehydrogenase (GAPDH) as a non-regulated reference gene. Gene expression at P2-P5 is reported as fold change compared to the gene expression at P1. Analyses of RT-PCR results were performed using GenEx version 5.0.1 (MultiD Analyses AB, Göteborg, Sweden) based on methods described by Pfaffl [12]. Statistical significance was set at *P* < 0.05. One-way ANOVA (ordinary one-way ANOVA with Sidak’s mutlitple comparisons test for normally distributed data; Kruskal-Wallis ANOVA with Dunn’s multiple comparisons test for non-normally distributed data) was used to compare expression of genes at P2-P5 to expression at P1 using Prism® 6.0 software (GraphPad Software Inc., San Diego, CA). Two-way ANOVA was used to determine if gene expression patterns over increasing cell passage were different between BECs from asthmatic and healthy subjects. Statistical analyses of clinical and lung function data were also performed using Prism®.

## Results

Bronchial brushings were obtained from both asthmatic (*n* = 6) and healthy donors (*n* = 6) to generate ALI cultures used in this study. Clinical characteristics for each group are shown in Table 1. Asthmatic and healthy donors were similar in age (11.16 ± 3.7 years vs. 11.98 ± 5.1 years, respectively, *p =* NS). There was a 2/3 male predominance with both groups (no difference between asthma vs. healthy). All asthmatic subjects displayed atopic features including eczema (50%), allergic rhinitis (83%), or positivity to aeroallergen by RAST IgE testing (83%). No history of eczema or allergic rhinitis was reported in the non-asthmatic group. One subject in the healthy group did display a positive RAST result. The majority of asthmatic subjects were using inhaled corticosteroids (83%) at the time of enrollment. There was a non-significant trend toward higher FENO levels in asthmatic subjects compared to healthy subjects (31.2 ± 24.2 ppb vs. 12.0 ± 5.1 ppb, *p* = 0.26). Total serum IgE levels were not significantly different between asthmatic and healthy subjects (306.5 ± 387.32 IU/mL vs. 185.83 ± 403.47 IU/mL, *p* = 0.6). Measures of lung function by spirometry demonstrated no differences in FVC or FEV_1_ between the groups; however, FEF_25-75_ (78.4% ± 4.9% vs 103.9% ± 5.5%, *p* = 0.03) and FEV_1_/FVC (0.78% ± 0.03% vs. 103.9% ± 5.5%, *p* = 0.02) were significantly lower in the asthmatic group and consistent with airway obstruction.

**Table 1:** Subject Characteristics

	Asthmatic Subjects	Healthy Subjects	*p* Value
	*n = 6*	*n = 6*	
Age yrs. (mean ± SD)	11.16 (3.7)	11.98 (5.1)	0.76
Sex (female/male)	4/6	4/6	
Currently using daily asthma controller (%)	5 (83%)	N/A	
History of atopy, *n*; (%)	6 (100%)	1 (17%)	< 0.01
Positive RAST, *n*; (%)	5 (83%)	1 (17%)	0.02
IgE IU/mL (median ± SD)	306.5 (387.32)	185.83 (403.47)	0.6
FVC % predicted (mean ± SD)	106.4 (13.1)	95.0 (13.9)	0.22
FEV_1_/FVC Ratio (mean ± SD)	0.78 (0.03)	0.89 (0.07)	0.02
FEV_1_% predicted (mean ± SD)	94.4 (14.1)	97.25 (13.9)	0.77
FEF_25-75_% predicted (mean ± SD)	78.4 (4.9)	103.9 (5.5)	0.03
FE_NO_ ppb (mean ± SD)	31.2 (24.2)	12.0 (5.1)	0.26

*RAST* Radioallergosorbent testing, *FVC* Forced vital capacity, *FEV*_*1*_ Forced expiratory volume in one second, *FEF*_*25-75*_ Forced expiratory flow between 25 and 75% of expiration

**Fig. 2:**
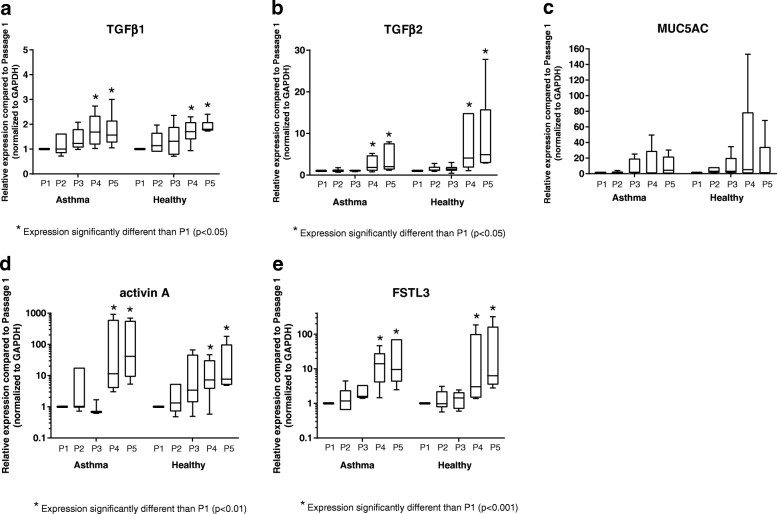
Expression of genes related to airway remodeling by primary BECs. Expression of TGFβ1 (**a**), TGFβ2 (**b**), MUC5AC (**c**), activin A (**d**), and FSTL3 (**e**) by BECs at P1 (*n* = 6 asthma donors, *n* = 6 healthy donors), P2 (*n* = 6 asthma donors, *n* = 6 healthy donors), P3 (*n* = 4 asthma donors, *n* = 6 healthy donors), P4 (*n* = 6 asthma donors, *n* = 6 healthy donors), and P5 (*n* = 6 asthma donors, *n* = 6 healthy donors) are presented as box-and-whisker plots which depict the interquartile range and median (the ends of each box represent the upper and lower quartiles, error bars represent the maximum and minimum, and the horizontal line within the box represents the median). To compare expression of genes at P2-P5 to expression at P1, and to compare patterns of gene expression between asthmatic and healthy donors, ordinary two-way ANOVA with Dunnett’s multiple comparisons test was used for normally distributed data, and Kruskal-Wallis ANOVA with Dunn’s multiple comparisons test was used for non-normally distributed data

Expression of genes associated with airway remodeling by differentiated primary BECs over successive passages (P1-P5) is depicted in Fig. 2. TGFβ1 expression was not significantly different from P1 through P3; however, expression was increased in P4 and P5 compared to P1 BECs from both asthmatic and healthy donors (*p* < 0.05, Fig. 2a), and there was not a difference in the pattern of expression with increasing passage between BECs from asthmatic and healthy subjects (*p* = 0.9). TGFβ2 expression was also not significantly different from P1 through P3, but similar to TGFβ1, expression was significantly increased in both P4 and P5 compared to P1 among both asthmatic and healthy subjects (*p* < 0.05, Fig. 2b), without significant differences in the pattern of gene expression by BECs from the two subject groups (*p* = 0.08). Expression of MUC5AC displayed marked variability beginning with P3, with the greatest variability observed at P4 and P5. However, given the high degree of variability, MUC5AC expression was not significantly different at P2-P5 compared to P1 (*p* = *0.3,* Fig. 2c) by BECs, nor was there a difference in the pattern of gene expression by BECs from asthmatic and healthy donors (*p* = 0.4). Gene expression of both activin A and FSTL3 were orders of magnitude greater at P4 and P5 compared to expression at P1 (*p* < 0.01 and *p* < 0.001, respectively) for BECs from both asthmatic and healthy donors, without pattern differences between the two subject groups (activin A: *p* = 0.08; FSTL3: *p* = 0.3); however, expression for both were not significantly different at P2 or P3 compared to P1 (Fig. 2d and e). Although the study was not designed or powered to assess differences in the expression of specific genes between asthmatic and healthy BECs, at P1 expression of TGFβ2 and MUC5AC, normalized to GAPDH, were significantly greater by asthmatic as compared to healthy BECs (Additional file 2: Figure S2).

**Fig. 3:**
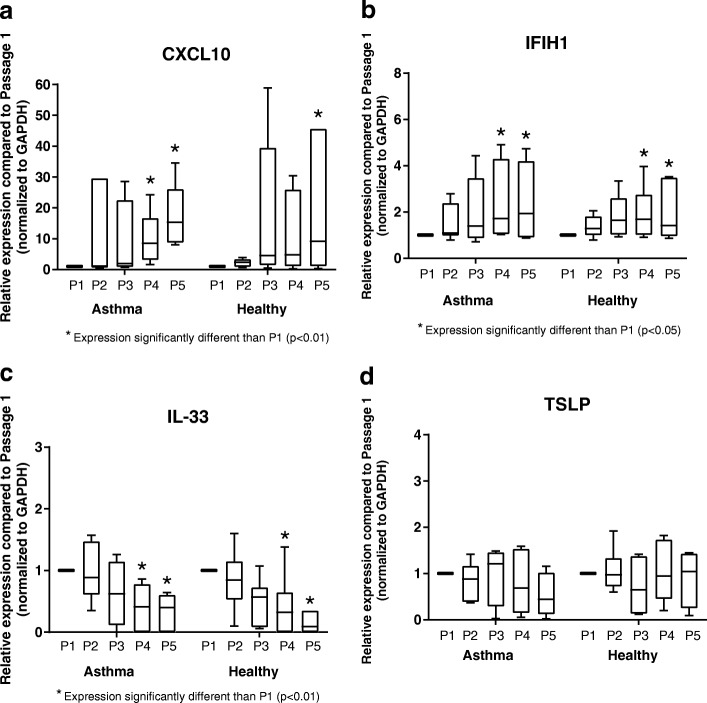
Expression of innate immunity and immunomodulatory genes by primary BECs. Expression of CXCL10 (**a**), IFIH1 (**b**), IL-33 (**c**), and TSLP (**d**) by BECs at P1 (*n* = 6 asthma donors, *n* = 6 healthy donors), P2 (*n* = 6 asthma donors, *n* = 6 healthy donors), P3 (*n* = 4 asthma donors, *n* = 6 healthy donors), P4 (*n* = 6 asthma donors, *n* = 6 healthy donors), and P5 (*n* = 6 asthma donors, *n* = 6 healthy donors) are presented as box-and-whisker plots which depict the interquartile range and median (the ends of each box represent the upper and lower quartiles, error bars represent the maximum and minimum, and the horizontal line within the box represents the median). To compare expression of genes at P2-P5 to expression at P1, and to compare patterns of gene expression between asthmatic and healthy donors, ordinary two-way ANOVA with Dunnett’s multiple comparisons test was used for normally distributed data, and Kruskal-Wallis ANOVA with Dunn’s multiple comparisons test was used for non-normally distributed data

In addition to genes associated with airway remodeling, expression of several genes involved in innate immune response (IFIH1, CXCL10) and immunomodulation (TSLP, IL-33) were also analyzed over increasing passages by BECs. There was significant variability in CXCL10 expression by BECs from both asthmatic and healthy donors from P2-P4 compared to expression at P1, with significantly increased expression at P4 and P5 compared to P1 by asthmatic BECs and significantly increased expression at P5 by healthy BECs (Fig. 3a), however, there was not a difference in the overall pattern of CXCL10 expression with increasing cell passage between asthmatic and healthy donors (*p* = 0.9). Expression of IFIH1 was significantly elevated at P4 and P5 compared to expression at P1 for BECs from both asthmatic and healthy donors (*p* < 0.05, Fig. 3b), without pattern differences between the subject groups (*p* = 0.4), but was not significantly different at P2 or P3. In contrast, Expression of IL-33 was significantly decreased at P4 and P5 compared to P1 by BECs from both asthmatic and healthy donors (*p* < 0.01; Fig. 3c); however, expression of IL-33 at P2 and P3 were not significantly different compared to P1, and there were no significant differences in IL-33 gene expression patterns with increasing cell passage between BECs from asthmatic and healthy donors (*p* = 0.4). Gene expression of TSLP remained stable throughout all 5 successive passages and was not significantly different compared to P1 by BECs from both asthmatic and health donors (Fig. 3d). Of note, at P1 expression of TSLP, normalized to GAPDH, was significantly greater by asthmatic as compared to healthy BECs (Additional file 2: Figure S2).

**Fig. 4:**
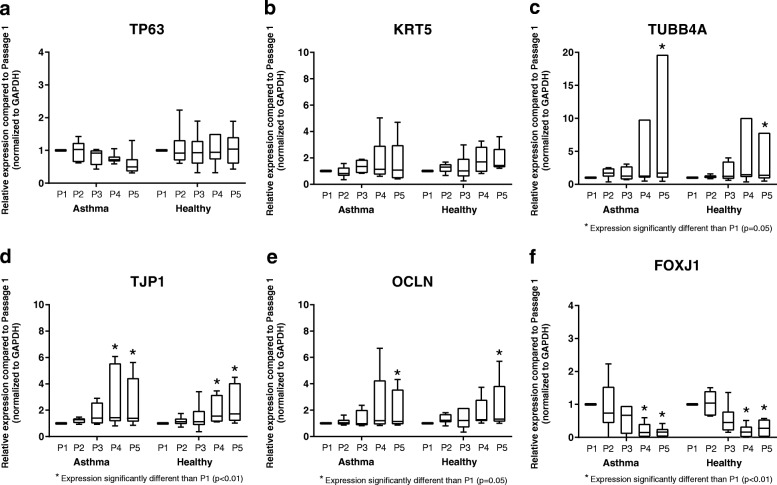
Expression of genes associated with airway epithelial basal cells, ciliogenesis, and epithelial tight junctions. Expression of TP63 (**a**), KRT5 (**b**), TUBB4A (**c**), TJP1 (**d**), OCLN (**e**), and FOXJ1 (**f**) by BECs at P1 (*n* = 6 asthma donors, *n* = 6 healthy donors), P2 (*n* = 6 asthma donors, *n* = 6 healthy donors), P3 (*n* = 4 asthma donors, *n* = 6 healthy donors), P4 (*n* = 6 asthma donors, *n* = 6 healthy donors), and P5 (*n* = 6 asthma donors, *n* = 6 healthy donors) are presented as box-and-whisker plots which depict the interquartile range and median (the ends of each box represent the upper and lower quartiles, error bars represent the maximum and minimum, and the horizontal line within the box represents the median). To compare expression of genes at P2-P5 to expression at P1, and to compare patterns of gene expression between asthmatic and healthy donors, ordinary two-way ANOVA with Dunnett’s multiple comparisons test was used for normally distributed data, and Kruskal-Wallis ANOVA with Dunn’s multiple comparisons test was used for non-normally distributed data

To assess the stability of expression of airway epithelial differentiation-associated genes and markers of epithelial basal cells over serial BEC passages, we measured the expression of the basal cell-associated gene TP63, the epithelial marker cytokeratin 5 (KRT5), ciliogenesis-associated genes TUBB4A and FOXJ1, and genes coding for the tight junctional proteins zona occluden-1 (TJP1) and occludin (OCLN). Expression of all of these genes was stable through at least P3 (Fig. 4). Expression of TP63 and KRT5 at passages 4 and 5 were not significantly different from expression at P1 by BECs from both asthmatic and healthy donors, but was more variable between individual cell lines at later passages (Fig. 4a, b). Among BECs from both asthmatic and healthy subjects expression of TJP1 was significantly greater at P4 and P5 as compared to P1 (*p* < 0.01; Fig. 4d), expression of TUBB4A and OCLN were significantly greater at P5 as compared to P1 (*p* = 0.05; Fig. 4c, e), and there were not significant differences in the overall patterns of expression of these genes with increasing cell passage between the asthmatic and healthy subject groups (TJP1: *p* = 0.5; TUBB4A: *p* = 0.6; OCLN: *p* = 0.8). In contrast to the other studied genes associated with epithelial differentiation, expression of FOXJ1 decreased in later passage ALI cultures, and expression was significantly lower at P4 and P5 compared to expression at P1 by BECs from both asthmatic and healthy BECs (*p* < 0.01; Fig. 4f), without pattern differences between the subject groups (*p* = 0.2). Although the study was not powered to assess differences in the expression of specific differentiation-associated genes or markers of epithelial basal cells between asthmatic and healthy BECs, no significant differences were observed at P1 (Additional file 2: Figure S2).

In order to assure that differences in gene expression patterns were not related to differential expression of housekeeping genes over multiple passages we compared the expression of GAPDH by BECs across the five passages and found no differences in mRNA Ct values (Additional file 3: Figure S1). We also assessed mRNA expression for each of the genes studied prior to normalization by GAPDH to show the natural variation in mRNA levels across asthmatic and healthy donors. For TGFβ1, TGFβ2, MUC5AC, FSTL3, and activin A, mRNA Ct values were significantly lower (greater un-normalized gene expression) at P4 and P5 compared to P1 (*p* < 0.05; Additional file 4: Figure S3) by BECs from both asthmatic and healthy donors, and there were not significant pattern differences in mRNA Ct values between the two subject groups with increasing cell passage for TGFβ1 (*p* = 0.4), FSTL3 (*p* = 0.4), and activin A (*p* = 0.6). However, mRNA Ct values were significantly lower (greater gene expression prior to normalization) by BECs from asthmatic as compared to healthy donors for TGFβ2 (*p* = 0.004) and MUC5AC (*p* = 0.04). Un-normalized mRNA Ct values for CXCL10 and IFIH1 were significantly lower at P4 and P5 compared to P1 among asthmatic and healthy BECs (*p* < 0.05; Additional file 5: Figure S4), whereas IL-33 mRNA Ct values were significant higher at P4 and P5 compared to P1 among asthmatic and healthy BECs (*p* < 0.05). For TP63 and KRT5, there were no significant differences in mRNA Ct values among or between asthmatic and healthy BECs with increasing cell passage (Additional file 6: Figure S5), whereas mRNA Ct values were significantly lower at P4 and P5 compared to P1 for asthmatic and healthy BECs for TUBB4A (*p* = 0.03), TPJ1 (*p* < 0.01), and OCLN (*p* < 0.05) without pattern differences between asthmatic and healthy BECs. Finally, FOXJ1 mRNA Ct values were significantly higher at P4 and P5 compared to P1 for asthmatic and healthy BECs, with a similar pattern between asthmatic and healthy BECs.

## Discussion

In the present study, we have demonstrated that primary differentiated BECs obtained from children with or without atopic asthma maintain stable expression of a panel of genes related to airway remodeling, innate immunity, immunomodulation, epithelial differentiation, and epithelial basal cells through passage 3 in ex vivo ALI cell cultures. We further report that in primary BECs beyond passage 3 expression of the studied genes became significantly more variable with expression of most genes increasing (TGFβ1, TGFβ2, activin A, FSTL3, MUC5AC, CXCL10, IFIH1, TUBB4A, TJP1, OCLN) and expression of other genes decreasing (IL-33, FOXJ1). Some genes were also found to be stable throughout the five passages studied (TSLP, TP63, KRT5). While the assumption that primary BECs retain their original phenotype up to passage 3 has been reported previously [4], those observations were based on a review of the available literature. In the present study, we present for the first time a study designed to compare the stability of gene expression of multiple airway remodeling, innate immunity, immunomodulation, epithelial differentiation, and epithelial basal cells genes by BECs over successive passages. Our findings further support the use of primary BECs obtained from pediatric donors at passage ≤3.

Primary ex vivo cell cultures of BECs obtained by bronchial brushings have been used successfully in children and adults for more than a decade and have become an attractive model to study the airway epithelium in various diseases. This is especially true in the pediatric population where obtaining cells via airway biopsies is problematic given that performing a sedated bronchoscopy in a pediatric subject for research purposes is ethically challenging. The initial description of the procedure to obtain primary BECs for research purposes from children already under anesthesia for a clinical indication was published by Doherty et al. in 2003 [7]. In that study, the authors compared the yield and viability of cells obtained via non-bronchoscopic airway brushings through an endotracheal tube under general anesthesia in 63 pediatric subjects to brushings obtained from a control population of adult patients undergoing bronchoscopy. Doherty and colleagues reported that a similar number of cells were obtained via the blind bronchial brushing as compared to the brushings obtained during bronchoscopy. Furthermore, the success rate of cell cultures obtained using this methodology was reported to be 82% despite a trend to lower viability of the cells obtained via the blind bronchial brushings, suggesting that this method harvested a sufficient amount of viable basal cells to establish the cell cultures [7]. Importantly, no adverse events where reported following the bronchial brushings and the authors concluded that this method of harvesting primary BECs in children was both effective and safe. In a separate study, Lane and colleagues reported similar results in a cohort of children with and without mild asthma [8]. In that study, the authors included an additional control group of children who did not undergo bronchial brushings and compared post-operative symptoms between the groups. No significant risk of adverse symptoms was reported with the most frequent symptom reported being a mild cough in less than half of the participants that underwent the bronchial brushings. Similar to the results reported by Doherty et al., Lane and colleagues found that the non-bronchoscopic brushings provided sufficient cells to carry out studies of RNA and protein-based assays, but also contained a sufficient population of basal cells to propagate in ex vivo cell cultures over multiple cell passages. Importantly, the subjects with mild asthma displayed no greater risk of adverse outcomes compared to the healthy control subjects further demonstrating the usefulness of this model in studying the role of BECs in pediatric asthma.

Optimal growth conditions for primary BECs have been extensively studied and have been reported elsewhere in the literature (reviewed by Gruenert et al. [13]). Most protocols utilize a defined, serum-free media that has been optimized to exclude contaminating cells such as fibroblasts. In addition to factors related to the growth media, the phenotype of the BECs grown in cell culture is critically dependent on the culture conditions. For example, cells can either be grown as a confluent monolayer in submerged cell culture or grown in a semi-permeable transwell insert at an air-liquid interface [13]. Cells grown at an ALI differentiate into a mucociliary phenotype that more closely resembles the native human airway epithelium than submerged cultures [14]. Recent studies have emphasized differences in epithelial responses during stimulation experiments based on whether a submerged vs. differentiated ALI culture model was used. For example, Kikuchi and colleagues compared the responses of BECs grown at an ALI to submerged BECs cultures. Following stimulation with IL-4 or IL-13 no differences in STAT6 phosphorylation were observed between BECs grown in submerged culture or at an ALI [15]. Conversely, the downstream effects of GM-CSF and TGFβ2 secretion in these models were found to be markedly different leading the authors to conclude that responses to IL-4 or IL-13 are critically dependent on the cell culture model system utilized in the study. In a separate series of experiments reported by Pezzulo et al., the authors examined the gene expression profile of primary BECs grown in either submerged conditions or differentiated at an ALI and compared their findings to both the in vivo condition as well as to a BEC cell line using genome-wide transcriptional profiling [16]. The authors of that study demonstrated that BECs differentiated at an ALI not only displayed morphological characteristics most similar to the in vivo condition (goblet cells, the presence of cilia, etc.), but also most closely recapitulated the transcriptional profile of the native airway epithelium. Thus, the authors concluded that the primary BECs differentiated at an ALI most closely represented the biology of the airway epithelium.

While primary BECs obtained via bronchial brushes or bronchoscopic biopsy differentiated at an ALI most closely resemble the in vivo airway epithelium, there are several caveats that must also be taken into account that may limit their utility in some experimental models. Given that primary BECs are derived from individual human donors there is a significant degree of variability between BECs from different donors. Furthermore, the findings in the present study would also suggest that cell passage number also contributes to increased phenotypic variability. The need to utilize greater replicates during experiments significantly increases the timeline and expense and may make primary cells less attractive for high-throughput screening studies. With this in mind, other studies have compared primary BECs differentiated at an ALI to existing transformed or immortalized cell lines. In one such study, Stewart and colleagues compared two different donor-derived primary BECs with three different available bronchial cell lines. In that study, the primary BECs expressed several markers of BEC differentiation and developed measurable trans-epithelial electrical resistance (TEER), albeit with intra-donor and intra-experimental variability [5]. Measurements of TEER were more consistent in Calu-3 cells; however, these cells also displayed distinct disparities in their expression of several markers of epithelial differentiation when compared to the primary BECs. Another cell line examined in that study (BEAS-2B) failed to differentiate at an ALI. These findings underscore the importance of choosing an appropriate model system for a given experimental question. Details regarding characteristics of available transformed cell lines have been reviewed elsewhere by Papazian et al. [17].

In this study we assessed over serial cell passages in ALI cultures the stability of expression of genes associated with airway epithelial basal cells and structural features unique to differentiated airway epithelium. Interestingly, expression of the airway epithelial basal cell associated gene p63 [18, 19] and epithelial marker cytokeratin 5 [20] were overall stable in ALI cultures over 5 consecutive cell passages, although there was a non-significant modest trend toward decreased and more variable p63 expression at P5 and increased variability of cytokeratin 5 expression at P4 and P5. Expression of the cillogenesis-associated genes TUBB4A and FOXJ1 [21, 22] as well as the tight junctional-associated genes TJP1 and OCLN [23] were stable through P3, however, expression of both of these genes become significantly more variable at passages beyond P3. In summary, similar to our observations for the remodeling-associated genes, innate immune response genes, and immunomodulatory genes studied, expression of several genes associated with airway epithelial differentiation were stable through P3, however, for later passages became significantly more variable.

The present study design includes several important strengths, including the use of primary BECs obtained from asthmatic and non-asthmatic children that are differentiated at an ALI. Additionally, our cohort is carefully phenotyped based on medical history and clinical features such as lung function and allergy testing. Despite these strengths our study also has several inherent limitations. In this study, our main outcome is the stability of gene expression over 5 successive passages in cell culture. We included cells obtained from both asthmatic and healthy donors in order to ensure that stability of gene expression was generalizable to both groups. Indeed, we have demonstrated that BECs obtained from both asthmatic and non-asthmatic donors similarly display stable gene expression through passage 3. Variability in gene expression beyond passage 3 was also observed in BECs derived from asthmatic and healthy donors to a similar degree. Although we did not observe statistically significant differences in the stability and variability of gene expression between BECs from asthmatic and healthy donors, our sample size was insufficient to detect subtle differences in patterns of gene expression with increasing passage between BECs from asthmatic and healthy children. Furthermore, this study is also underpowered to perform subgroup analysis of the data such as gender differences. Lung function data demonstrates that our cohort of asthmatic donors have a mild degree of airflow limitation signifying a relatively mild asthma phenotype in our cohort.

Additional limitations of our study include that we did not perform TEER measurements over serial passages and did not perform histological sections and/or immunostaining of our ALI cultures for basal cell markers or proteins associated with airway epithelial cell differentiation. Although beyond the scope of the current study, such outcome measures would be of interest in future studies of primary airway epithelial cells over serial culture passages. We did however study the expression stability of genes associated with airway epithelial basal cells, ciliogenesis, and epithelial tight junctions. Expression of the basal cell-associated gene TP63 as well as cytokeratin 5 were stable through P5, whereas expression of genes associated with ciliogenesis and tight junctions were less stable beyond P3. Of note, several groups have demonstrated over the past several years that airway epithelial basal cells can be expanded in culture and retain their ability to differentiate at the ALI many passages removed from the host [21, 24]. A final limitation of our study is that we did not analyze airway epithelial gene expression across serial passages from submerged undifferentiated cultures.

## Conclusions

While ex vivo primary BECs differentiated at an ALI represent one of the best available models to study the role of the airway epithelium in disease processes such as asthma in children, care must be taken to ensure that cell phenotype and gene expression patterns are preserved such that ex vivo studies reflect the in vivo condition as closely as possible. We have provided new evidence that primary BECs from children differentiated at an ALI display stable gene expression patterns over 3 successive passages; however, we have also shown that gene expression becomes significantly more variable at later passages, which could potentially affect study outcomes if later passages are used in experiments. These findings should be carefully considered in future study designs using primary BECs in ex vivo model systems.

## Additional files

Additional file 1 Primary quantitative PCR datasets. (XLSX 37 kb)

Additional file 2: **Figure S2.** Comparison of gene expression between asthmatic and healthy BECs at passage 1 (P1). Expression of genes related to airway remodeling (panel A.; TGFβ1, TGFβ2, MUC5AC, activin A, and FSTL3), innate immunity and immunomodulatory genes (panel B.; CXCL10, IFIH1, IL-33, and TSLP), and expression of genes associated with airway epithelial basal cells, ciliogenesis, and epithelial tight junctions (panel C.; TP63, KRT5, TUBB4A, TJP1, OCLN, and FOXJ1) by primary asthmatic (grey plots) and healthy (white plots) BECs at P1 (*n* = 6 asthma donors, *n* = 6 healthy donors). Expression of each gene (normalized to GAPDH) relative to the median of healthy BECs are presented as box-and-whisker plots which depict the interquartile range and median (the ends of each box represent the upper and lower quartiles, error bars represent the maximum and minimum, and the horizontal line within the box represents the median). The Wilcoxon signed rank test was used to test differences in expression of specific genes between asthmatic and healthy BECs. (EMF 109 kb)

Additional file 3: **Figure S1.** Expression of GAPDH as a reference gene. Ct values for GAPDH were compared for each cell passage. No significant differences were observed from P1 through P5. (TIF 73 kb)

Additional file 4: **Figure S3.** Un-normalized mRNA expression of genes related to airway remodeling by primary BECs. Un-normalized mRNA Ct values for TGFβ1 (A.), TGFβ2 (B.), MUC5AC (C.), activin A (D.), and FSTL3 (E.) by BECs at P1 (*n* = 6 asthma donors, *n* = 6 healthy donors), P2 (*n* = 6 asthma donors, *n* = 6 healthy donors), P3 (*n* = 4 asthma donors, *n* = 6 healthy donors), P4 (*n* = 6 asthma donors, *n* = 6 healthy donors), and P5 (*n* = 6 asthma donors, *n* = 6 healthy donors) are presented as individual data points for each donor cell line. To compare expression of genes at P2-P5 to expression at P1, and to compare patterns of gene expression between asthmatic and healthy donors, ordinary two-way ANOVA with Dunnett’s multiple comparisons test was used for normally distributed data, and Kruskal-Wallis ANOVA with Dunn’s multiple comparisons test was used for non-normally distributed data. (EMF 195 kb)

Additional file 5: **Figure S4.** Un-normalized mRNA expression of innate immunity and immunomodulatory genes by primary BECs. Un-normalized mRNA Ct values for CXCL10 (A.), IFIH1 (B.), IL-33 (C.), and TSLP (D.) by BECs at P1 (*n* = 6 asthma donors, *n* = 6 healthy donors), P2 (*n* = 6 asthma donors, *n* = 6 healthy donors), P3 (*n* = 4 asthma donors, *n* = 6 healthy donors), P4 (*n* = 6 asthma donors, *n* = 6 healthy donors), and P5 (*n* = 6 asthma donors, *n* = 6 healthy donors) are presented as individual data points for each donor cell line. To compare expression of genes at P2-P5 to expression at P1, and to compare patterns of gene expression between asthmatic and healthy donors, ordinary two-way ANOVA with Dunnett’s multiple comparisons test was used for normally distributed data, and Kruskal-Wallis ANOVA with Dunn’s multiple comparisons test was used for non-normally distributed data. (EMF 155 kb)

Additional file 6: **Figure S5.** Un-normalized mRNA expression of genes associated with airway epithelial basal cells, ciliogenesis, and epithelial tight junctions by primary BECs. Un-normalized mRNA Ct values for TP63 (A.), KRT5 (B.), TUBB4A (C.), TPJ1 (D.), FOXJ1 (E.), and OCLN (F.) by BECs at P1 (*n* = 6 asthma donors, *n* = 6 healthy donors), P2 (*n* = 6 asthma donors, *n* = 6 healthy donors), P3 (*n* = 4 asthma donors, *n* = 6 healthy donors), P4 (*n* = 6 asthma donors, *n* = 6 healthy donors), and P5 (*n* = 6 asthma donors, *n* = 6 healthy donors) are presented as individual data points for each donor cell line. To compare expression of genes at P2-P5 to expression at P1, and to compare patterns of gene expression between asthmatic and healthy donors, ordinary two-way ANOVA with Dunnett’s multiple comparisons test was used for normally distributed data, and Kruskal-Wallis ANOVA with Dunn’s multiple comparisons test was used for non-normally distributed data. (EMF 224 kb)

## Abbreviations

ALI: Air-liquid interface
BDR: Bronchodilator responsive
BEC: Bronchial epithelial cell
BEGM: Bronchial epithelial growth medium
FEF_25-75_: Forced expiratory flow between 25 and 75% of FVC
FENO: Fraction of exhaled nitric oxide
FEV1: Forced expiratory volume in 1 s
FVC: Forced vital capacity
GAPDH: Glyceraldehyde 3-phosphate dehydrogenase
ICS: Inhaled corticosteroid
IgE: Immunoglobulin E
RAST: Radioallergosorbent testing
RNA: Ribonucleic acid
