# Supplementary figures and images for: Stability of gene expression by primary bronchial epithelial cells over increasing passage number
Source: BMC Pulm Med. 2018 May 29;18:91. doi: 10.1186/s12890-018-0652-2 (PMC5975426; doi:10.1186/s12890-018-0652-2)

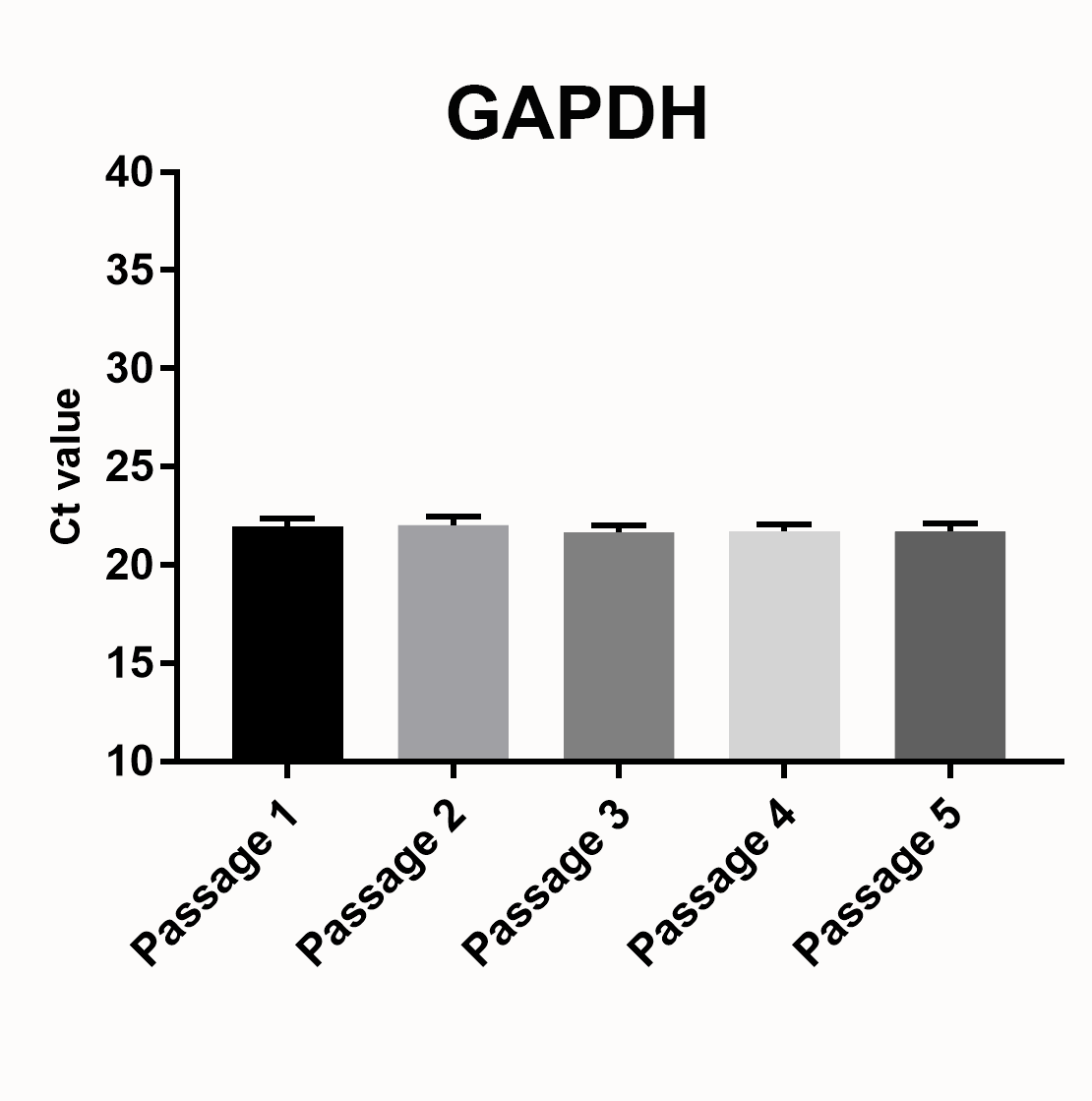

Supplement: Supplementary file 3 — Figure S1. Expression of GAPDH as a reference gene. Ct values for GAPDH were compared for each cell passage. No significant differences were observed from P1 through P5. (TIF 73 kb) [file 12890_2018_652_MOESM3_ESM.tif]
